# Adoptive T-cell therapies to overcome T cell-dependent immune dysregulations in COVID-19

**DOI:** 10.3906/biy-2109-85

**Published:** 2021-12-20

**Authors:** Sevgi KALKANLI TAŞ, Merve Saide UZUNOĞLU, Aylin Seher UZUNOĞLU, Duygu KIRKIK, Derya ALTUNKANAT, Nevin KALKANLI

**Affiliations:** 1 Department of Immunology, Hamidiye Medicine Faculty, University of Health Sciences, İstanbul, Turkey; 2 Department of Immunology, Hamidiye Institute of Health Sciences, University of Health Sciences, İstanbul, Turkey; 3 Department of Medical Biology, Hamidiye Medicine Faculty, University of Health Sciences, İstanbul, Turkey; 4 Diyarlife Hospital, Department of Dermatology, Diyarbakır, Turkey

**Keywords:** Adoptive cell therapy, COVID-19, immunotherapy, SARS-CoV-2, T cell response

## Abstract

Coronavirus disease 2019 (COVID-19) pandemic has been an important global interest that affected millions of people, and it requires a deep investigation of the disease immunology for developing further therapeutic applications. Adoptive T cell therapy promises to address T cell-dependent immune dysregulation in COVID-19 patients by the generation of specific T cell clones against virus-specific antigens. Additionally, targeting B cell-dependent protection through COVID-19 vaccines, which have been developed in the recent year, possessed sufficient prevention for spreading the virus, since the cases and deaths related to COVID-19 tend to decrease after the vaccination. However, adoptive cell therapies are now encouraging scientists to deal with pathological challenges like inadequate T cell-dependent immune response or lymphopenia, since they are the most frequent outcome of severe infection, especially in immunocompromized patients. In this review, the current knowledge of immunopathology of COVID-19 was aimed to be highlighted along with the T cell responses against SARS-CoV-2 to comprise a basis for therapeutics. Moreover, current therapeutics and treatment strategies for COVID-19 were discussed to evaluate possible agents. Furthermore, the use of adoptive T cell therapy representing an emerging therapeutic approach was purposed to be presented comprehensively against SARS-CoV-2 infection. Even though further studies are needed to fully understand T cell response against SARS-CoV-2 in order to develop therapies to provide long term and efficient protection, adoptive cell therapies now meet the demand for a large population of people who suffer immunocompromization, considering the previous usage of the technique for different infectious diseases.

## 1. Introduction

Coronavirus disease 2019 (COVID-19) has become the biggest burden to the world in terms of health and economy as the third coronavirus outbreak of the 21st century because of a positive sense, single-stranded RNA virus called Severe Acute Respiratory Syndrome Coronavirus 2 (SARS-CoV-2) was detected in over 249 million confirmed cases worldwide as of November 9, 2021. The disease was firstly raised in the city of Wuhan in China in December 2019, and the number of deaths caused by the tremendous spread of the virus hit 5 million all over the world according to the data of the World Health Organization (WHO) (World Health Organization, 2021).

The virus has now been circulated massively around the world through human-to-human contact and became a big burden to healthcare professionals (Kumar and Al Khodor, 2020). Infected patients developed very critical diseases and poor clinical outcomes including respiratory failure, fever, cough, systemic shock, multi-organ failure, and acute respiratory distress syndrome (ARDS) with severe pneumonia and need in-patient care and mechanical ventilation (Blanco-Melo et al., 2020; Kumar and Al Khodor, 2020). The severe inflammation in response to SARS-CoV-2 has been observed mainly in critical cases, which is presumably the most involved mechanism for the pathogenesis of COVID-19 including an excessive amount of inflammatory cytokine and chemokine release, prolonged and serious lymphopenia, and tissue infiltration of inflammatory cells (Sengupta, 2020). Several vaccines developed with different techniques are now encouraging scientists; however, further investigation is required for people who need treatment for ongoing SARS-CoV-2 infection, since vaccines are only effective for SARS-CoV-2-negative individuals. Therefore, revealing the pathophysiology and the underlying mechanisms of COVID-19 are of high importance in terms of developing new treatment strategies like antiviral drugs, effective vaccine candidates, and inhibitors targeting both viral spike proteins and host receptors. Among those strategies, focusing on the main concern in severe cases about the mechanism of clearance of the infection, which is employed fundamentally by T cells, could be a rational approach by implementing adoptive T cell therapy.

## 2. Pathophysiology and clinical presentation of COVID-19

The infection of the airborne virus SARS-CoV-2 occurs via droplets or contacts that mediate virus entry to the body and ultimately to cells (Parasher, 2020; Chee et al., 2021). Betacoronaviruses utilize this cell entry mechanism, which is a fundamental element for interspecies transmission, through their surface spike glycoprotein binding to the receptor of the transmitted cell (Zhao et al., 2020;To et al., 2021). The spike glycoprotein is a trimer projecting out of the virus and involves two subunits, in which recognition and binding take place, the S1 subunit, and cleavage, and release the spike fusion protein through the S2 (Kumar and Al Khodor, 2020; Parasher, 2020; Sardar et al., 2020; Sridhar and Nicholls, 2021; To et al., 2021). As Middle East Respiratory Syndrome Coronavirus (MERS-CoV) enters its target cell via the dipeptidyl peptidase-4 (DPP4) receptor, SARS-CoV and SARS-CoV-2 both have a cell entering machinery through binding angiotensin-converting enzyme 2 (ACE2), which is the most characterized receptor for these viruses (Letko et al., 2020; Millet et al., 2021). The spike protein, which all betacoronaviruses encode, contains a very specific region known as the receptor-binding domain (RBD) that binds to the cell receptor of the host and mediates viruses to enter the cell (Crooke et al., 2020). Following attachment of the RBD of spike protein on the S1 subunit to its receptor ACE2, viral entry involves the cleavage of spike protein with the host transmembrane protease serine 2 (TMPRSS2) so that the fusion peptide releases from the S2 subdomain, and the virus enters the cell by endocytosis (Johansen et al., 2020; Kumar and Al Khodor, 2020; Parasher, 2020; Wiersinga et al., 2020; Zhu et al., 2020).

ACE2 is a well-defined integral membrane metalloproteinase involving in the renin-angiotensinaldosterone system (RAAS) and is highly abundant throughout the body (Barker and Parkkila, 2020; Bourgonje et al., 2020; Tas et al., 2020; Tay et al., 2020). Both ACE2 and TMPRSS2 are especially present on targeted alveolar epithelial type 2 cells in the human body (Wiersinga et al., 2020). Although ACE2 expressing cells can mainly facilitate cell entry for the virus, the revealed pathophysiological mechanisms of COVID-19 represent that not all organs share the same infection state and outcome, which may be due to different parameters affecting the receptor expression, disease severity, and progress (Bourgonje et al., 2020; Tas et al., 2020; Wiersinga et al., 2020).

ACE2 expression is the most abundant in type 2 alveolar cells, epithelial cells of the intestine, vascular endothelial cells, cardiac cells, and proximal tubular cells of the kidney (Bourgonje et al., 2020; Tas et al., 2020). Even though lungs are the main concern in COVID-19 patients, clinical outcomes showed that patients face serious tissue damages in their hearts and kidney as well (Bourgonje et al., 2020). The acquired virus through respiratory aerosols attaches to upper respiratory tract nasal epithelial cells where ACE2 is abundantly present. During the first few days, on-site replication and eventually a rapid increase in virus population occurs as well as increasing transmission through ciliated cells of airway epithelium. Reported cases predominantly do not develop further stages of the infection, as the immune response is qualified to control the propagated virus (Parasher, 2020; Stratton et al., 2021). However, progressing infection throughout alveolar epithelial type 2 cells of the lower respiratory tract becomes the Achilles heel for the immune system, as substantive immune response fails to exist, resulting in excessive cytokine and inflammatory components release from pneumocytes (Parasher, 2020; Chee et al., 2021; Sridhar and Nicholls, 2021). This trafficking of cytokines and chemokines attracts neutrophils, CD4+ T cells, and CD8+ T cells to the lung tissue in which gradually growing inflammation and tissue damage adds a greater complexity. The widespread alveolar damage emerges ultimately resulting in an ARDS related to chronic depredation caused by the accumulating cells and viral replication generating serious damage on type 2 as well as type 1 pneumocytes (Mathew et al., 2020; Parasher, 2020; Tay et al., 2020; Neurath, 2021; Sridhar and Nicholls, 2021; Stratton et al., 2021).

Within COVID-19 patients, prolonged inflammation against the virus is considered to be a remarkable factor for the progression of the disease and mortality, and it is associated with the elevated degree of cytokines in circulation, extreme lymphopenia, and severe penetration of mononuclear cells into the lungs, heart, kidneys, spleen and lymph nodes as well as significantly increased C-reactive protein (CRP), Pentraxin-3, serum ferritin, and D-Dimers (Felsenstein et al., 2020; Harrison et al., 2020; Merad and Martin, 2020; Brunetta et al., 2021; Genc AB et al., 2021).

## 3. T cell response in COVID-19

T cell immunity in COVID-19 remains under deep investigation (Luo et al., 2020; Mathew et al., 2020; Rodda et al., 2021; Toor et al., 2021) because viral infections require efficient T cell responses for complete clearance and long-term protection against viruses (Nelde et al., 2020; Agerer et al., 2021; Jarjour et al., 2021). Immune defense is initiated firstly through innate immune recognition finally leading to the stimulation of adaptive immune response, while the disease progresses and ultimately increases leukocyte activation (Dai et al., 2021; Rha et al., 2021; Sette and Crotty, 2021). The crucial step in the lymphocyte activation is the presentation of antigens by antigen-presenting cells (APCs) such as monocytes, macrophages, and dendritic cells that drive the induction of T cells and B cells leading to the elimination of infected cells by CD8+ T cells and generation of humoral response by B cells (Gujar et al., 2020; Dai et al., 2021; Rodda et al., 2021). Acknowledging the effector functions, follicular helper CD4+ T (Tfh) cells employ priming of B lymphocytes in order to generate high-affinity virus-specific neutralizing antibodies; besides, CD4+ T cells help CD8+ T cells to respond to chronic infections consequentially by producing IL-21, which is a cytokine of Tfh cells (Tay et al., 2020; Laidlaw et al., 2016; Cui et al., 2021; Jarjour et al., 2021; Sette and Crotty, 2021). Also, to promote the induction of the immune cells to the environment where the infection occurs, differentiated distinct subpopulations of CD4+ T helper (Th) cells are needed for cytokine production such as Th1, Th2, and Th17 cells (Swadling and Maini, 2020; Tay et al., 2020; de Candia et al., 2021; Sette and Crotty, 2021).

Mainly, CD8+ T cells are necessary to attack and destroy the infected cells directly by generating an antigen recognition against virus antigens presented on major histocompatibility complex (MHC) class-I molecules found on the infected cell surface (Swadling and Maini, 2020; Bertoletti et al., 2021; Rha et al., 2021; Toor et al., 2021). Cytotoxic molecules, including granzyme B, perforin, and IFN-γ are released by CD8+ T cells, and infected cells are eliminated through downstream mechanisms and pathways (Luo et al., 2020; Swadling and Maini, 2020; Toor et al., 2021). Finally, that recognition leads to the specialization and generation of immunological memory (Gujar et al., 2020; Tay et al., 2020; Bertoletti et al., 2021; Jarjour et al., 2021; Rodda et al., 2021). Furthermore, virus-derived peptide presentation to CD4+ T helper cells is executed by professional APCs through MHC class-II molecules once viral antigens are picked up from their surroundings and processed by APCs (Bertoletti et al., 2021; de Candia et al., 2021). Th cells polarize predominantly in the direction of Th1 when Th cells are subjected to the antigen presentation, resulting in the generation of IFN-γ and associated cytokines to remove the virus (Sette and Crotty, 2021; Toor et al., 2021).

There seems to be a significant confusion as to whether adaptive immune responses to SARS-CoV-2 are defensive or pathogenic, although both outcomes have been revealed since the beginning of the infection depending on the duration, structure, or degree of the adaptive immune response (Chen and John Wherry, 2020; Grifoni et al., 2020; Mathew et al., 2020). In the respiratory tract and other tissues, autopsies also showed elevated levels of SARS-CoV-2, indicating inadequate immune responses (Chen and John Wherry, 2020; Mathew et al., 2020). Furthermore, few reports demonstrated that an overstimulated immunity contributes to the pathophysiological outcome in which possible mechanisms have been mostly associated with exhaustion or impairment of T cells in the patients failing viral clearance (Mathew et al., 2020; Nelde et al., 2020; Rha et al., 2021; Toor et al., 2021). Nevertheless, it is still not clearly defined that immunopathology is observed because of whether over-activation or suppression of the adaptive immune response in serious cases of the SARS-CoV-2 infection (Agerer et al., 2021; de Candia et al., 2021; Jarjour et al., 2021; Toor et al., 2021). Correspondingly, both hyperactivation or exhaustion of T cells in patients with COVID-19 has been seen in several studies (Diao et al., 2020; Fathi and Rezaei, 2020; Mathew et al., 2020; Weiskopf et al., 2020; Rha et al., 2021; Toor et al., 2021).

To induce an adequate immune response, a defensive primary T cell response against SARS-CoV-2 infection involves the recruitment and activation of antigen-specific naïve CD4+ T cells and CD8+ T cells followed by expanding the cell population rapidly and differentiating into appropriate effector cell types (Jarjour et al., 2021). Most of the studies showed that T cells detect structural proteins of SARS-CoV-2 predominantly, notably spike, membrane, and nucleocapsid proteins. They appear to evoke a robust T-cell response in both symptomatic and asymptomatic people, and, therefore, analysis demonstrated that they are immunogenic for both CD4+ T cells and CD8+ T cells (Rydyznski Moderbacher C et al., 2020; Bertoletti et al., 2021; Bilich et al., 2021; Le Bert N et al., 2021).

SARS-CoV-2-specific CD4+ T cells showed a greater correlation with reduced severity of COVID-19 than antibodies and CD8+ T cells, while successful generation of SARS-CoV-2-specific CD4+ T cells at the early onset of the COVID-19 was correlated with increased viral clearance and, thus, moderate the disease (Rydyznski Moderbacher C et al., 2020; Sette and Crotty, 2021). Differentiation of SARS-CoV-2-specific CD4+ T cells into Th1 and Tfh cells in response to SARS-CoV-2 has been observed, and SARS-CoV-2 specific circulating Tfh cells, which are responsible for the generation of virus-specific neutralizing antibody response, and memory B cells were found to be accelerated in the acute phase of COVID-19 (Rydyznski Moderbacher C et al., 2020; de Candia et al., 2021; Jarjour et al., 2021; Sette and Crotty, 2021). On the other hand, the generation of Th2, Th17, and regulatory T cells from CD4+ T cells was associated with increasing disease severity according to CD8+ T cell exhaustion (Rydyznski Moderbacher C et al., 2020; Bertoletti et al., 2021; Jarjour et al., 2021; Sette and Crotty, 2021).

Prolonged T cell exhaustion is the characteristic of COVID-19, culminating in progressive immune deficiency, coinfection, and death as well. In a study that was conducted with 3 COVID-19 cases, bone marrow hypoplasia and reduced lymphocyte levels, degenerative changes, and necrosis of the spleen cell were pathologically observed (Luo et al., 2020; Yao et al., 2020). The depletion of T cells can be induced by either T cell hyperactivation or overexpression of proapoptotic proteins (eg, FAS or TRAIL) (Esmaeilzadeh and Elahi, 2021). T cell counts have steadily recovered the values similar to mild cases of patients suffering severe COVID-19 (Luo et al., 2020). The most important mechanism, which is developed in COVID-19 patients following therapy, is immune restoration, marked by an elevation in the number of T cells. Improving immune reconstruction, especially COVID-19 specific T cells, is, therefore, a significant research field (Luo et al., 2020).

Increased virus-infected cells contribute to tissue destruction and more fatal implications are compromised by viral progenies (Luo et al., 2020; Toor et al., 2021). CD4+ T cells and CD8+ T cells struggle to have sufficient cellular immune response followed by an insufficient humoral response to remove virus-infected cells under the conditions where the first line of defense was failed (Altmann and Boyton, 2020; Swadling and Maini, 2020; Toor et al., 2021). Nonetheless, priming of Th cells for the phenotype of Th17 is dominated under these circumstances, resulting in the suppressed immune responses mediated by Th1 in which cytokines derived from Th17 have been involved in the severe lung pathophysiology found in cases with ARDS by this way of suppression (Luo et al., 2020; Esmaeilzadeh and Elahi, 2021; Patel et al., 2021; Rodda et al., 2021; Toor et al., 2021). Cytokine production, particularly TNF-α, IL-1β, IL-2, IL-6, and IL-10, is elevated in extreme COVID-19 conditions, contributing to cytokine release syndrome formation, which causes more unfavorable outcomes and can ultimately lead to lymphopenia (Altmann and Boyton, 2020; Fathi and Rezaei, 2020; Luo et al., 2020; Mathew et al., 2020; Weiskopf et al., 2020; Patel et al., 2021; Toor et al., 2021). In COVID-19 cases, lymphopenia is an expected pattern, especially in elder people, which may be a crucial feature relevant to the seriousness of the disease and mortality (Luo et al., 2020). Targeted depletion of natural killer (NK) cells together with both CD4+ T cells and CD8+ T cells are observed in lymphopenia, and activation of T cells and elevated expression of inhibitory T cell receptors are exceedingly notable in severe cases than in moderate ones (Song et al., 2020; Toor et al., 2021). A large number of cytotoxic molecules are released from CD8+ T cells in patients with severe cases; however, the reason for lymphopenia is still unclear (Fathi and Rezaei, 2020; Song et al., 2020).

Formation of memory T cell response is required for prolonging protection against SARS-CoV-2, dependent on the generation of memory CD4+ T cells, which are essential for the induction of B cells and CD8+ T cells (Jarjour et al., 2021). Although recent studies revealed that SARS-CoV-2 specific T cell memory lasts longer than B cell memory, cross-reactive T cell responses constitute a relatively important section of the defense later on the possible intercourse with the virus (Agerer et al., 2021; Bilich et al., 2021; DiPiazza et al., 2021). Besides all, there has been a great deal of interest in the possibility of T cell cross-reactivity between human circulating common cold coronaviruses (229E, NL63, HKU1, and OC43) and SARS-CoV-2. Cross-reactive T cells that are already present in the body have the potential to accelerate viral elimination and enhance patient outcomes after infection (DiPiazza et al., 2021).

T cell immunity preserves its place as the most important line of response against SARS-CoV-2, especially for people who are failed to develop seroconversion and immunocompromized patients who have a lower possibility of developing an efficient antibody response (Gallagher et al., 2021). One study showed that IgG antibody titers can be prolonged up to one year after SARS-CoV-2 infection in which severe cases have even higher antibody titers through twelve months than nonsevere cases, yet gradually decreasing of IgG titers have been observed for the first six months up to 70% for all cases and twelve months up to 88% (Xiao et al., 2021). In the past few months, some studies evaluated the prolongation and protection status of antibody immune responses after vaccination, though several vaccine studies already revealed that antibody titers show a tendency to drop in 3 months after two doses of vaccination with RNA vaccine (BNT162b2) (Favresse et al., 2021). Scientists have been now considering booster shots to increase the antibody titers provided by the vaccines because antibody levels tend to decrease after the first two shots (Urbanowicz et al., 2021). While one study showed that a third dose of RNA vaccine (BNT162b2) as a booster increased the neutralizing antibody titers in the patients after two doses, another study with the inactive vaccine or recombinant RBD protein vaccine did not induce a boosting after two doses of inactive vaccine, and an adenovirus vaccine used as a booster showed induced higher T cell response (Zhang et al., 2021). Even though vaccine studies are still ongoing, other crucial factors alter the effect of antibody responses generated by vaccines such as the SARS-CoV-2 variant of concerns, which finally raise the question of whether humoral immunity could provide adequate long-term protection by natural immunization or vaccination (Bertoletti et al., 2021; Souza et al., 2021; Tauzin et al., 2021). The possible answer could be no, even though further studies are required for both B cells and T cells in which generation of adequate B cell response and memory formation is functionally dependent on the generation of adequate T cell response by whether natural infection or vaccination. Therefore, focusing on T cell response rather than humoral response would change the direction of the pandemic positively in terms of the generation of new therapeutics.

## 4. Current therapeutics and adoptive T-cell therapies against COVID-19

Current scientific studies predominantly focused on vaccine development for COVID-19 in order to prevent the spread of the infection and reduce the severity of the disease. Therefore, COVID-19 vaccines preserve their places as defensive measures in the health care system for uninfected patients although wide-spreading SARS-CoV-2 variant of concerns (VOCs) constitute several challenges concerning the protective level of the vaccines in terms of immune evasion (Edara et al., 2021; Garcia-Beltran et al., 2021; Shen et al., 2021; Wang P. et al., 2021b; Wang Z. et al., 2021). Moreover, Bates et al. (2021) revealed that age-dependent reduction was detected in neutralizing antibody titers of people who received two doses of BNT162b2 vaccine against the USA-WA1/2020 strain and the P.1 variant of concern (Bates et al., 2021). Also, the duration of the protection provided after infection or by vaccination could be insufficient to predict the long-term immunity yet and arise the questions for postvaccination that focused on whether people need vaccination at regular intervals (Bilich et al., 2021; Dan et al., 2021; Edara et al., 2021; Eyre et al., 2021; Gerhards et al., 2021; Goel et al., 2021; Knies et al., 2021; Wang Z et al., 2021) While further vaccine research has been considered for protection of people who have no existing infection, treatment strategies are on demand for those people with ongoing SARS-CoV-2 infection (Gavriatopoulou et al., 2020; Kaplon and Reichert, 2021; Phan et al., 2021).

Commonly used strategies for COVID-19 treatment focus on neutralizing the viral material using convalescent plasma or therapeutic monoclonal antibodies (mAbs), virus-host interaction blockers, replication and transcription complex blockers, and immunomodulators (Gavriatopoulou et al., 2020; Woo, 2021). Neutralizing mAb treatment is now encouraging scientists to treat infected patients although SARS-CoV-2 variants still possess an important line of obstacles (Wang P. et al., 2021a, 2021b). Nevertheless, none of the ongoing treatment options focus on the major challenge faced by the failure of the immune system during SARS-CoV-2 infection (Gavriatopoulou et al., 2020; Monzavi et al., 2021). In order to generate a long-term immunity and prime the effector cells that actually can clear the infection and possess a memory for the future, adoptive cell therapy (ACT) meets the demand for people who suffer failed conventional COVID-19 therapy because of a certain condition such as cancer, immunocompromizing, or old age (Keller et al., 2020; Bertoletti and Tan, 2020; Chan et al., 2021; Ferreras et al., 2021; Monzavi et al., 2021; Zmievskaya et al., 2021). As it is seen in the previously described study that measures the neutralizing antibody titers of people with USA-WA1/2020 strain and the P.1 variant of concern who received two doses of BNT162b2 vaccine, vaccine-induced neutralization antibody titer against SARS-CoV-2 decreased in an age-dependent manner.

While ACT already has been a popular method in the production of cancer therapies during the twenty-first century, viral infections have been attracted a great deal of attention to generating virus-specific T cells using ACT strategies (Gujar et al., 2020; MacKay et al., 2020; Bachanova et al., 2020; Dai et al., 2021; Ferreras et al., 2021). Recently, developing SARS-CoV-2-specific T cell-dependent therapies have gained a wide currency, since inducing persistent immune response and memory have been the main concern for COVID-19 (Keller et al., 2020; Bilich et al., 2021; Chan et al., 2021; Ferreras et al., 2021).

Generation of virus-specific T cells mainly aims to achieve destroying the infected cells by antigen-specific CD8+ T cells that release cytotoxic granules, while antigen-specific CD4+ T cells are generally employed by prime B cells to produce virus-specific antibodies as described earlier. Moreover, ACT also aims to generate both CD8+ T cells and CD4+ T cells secreting cytokines such as IFN-γ and TNF-α/β that employ communicating other immune system compartments during the viral infection (Bertoletti and Tan, 2020; Keller et al., 2020; Bilich et al., 2021; Chan et al., 2021; Ferreras et al., 2021).

**Figure 1:**
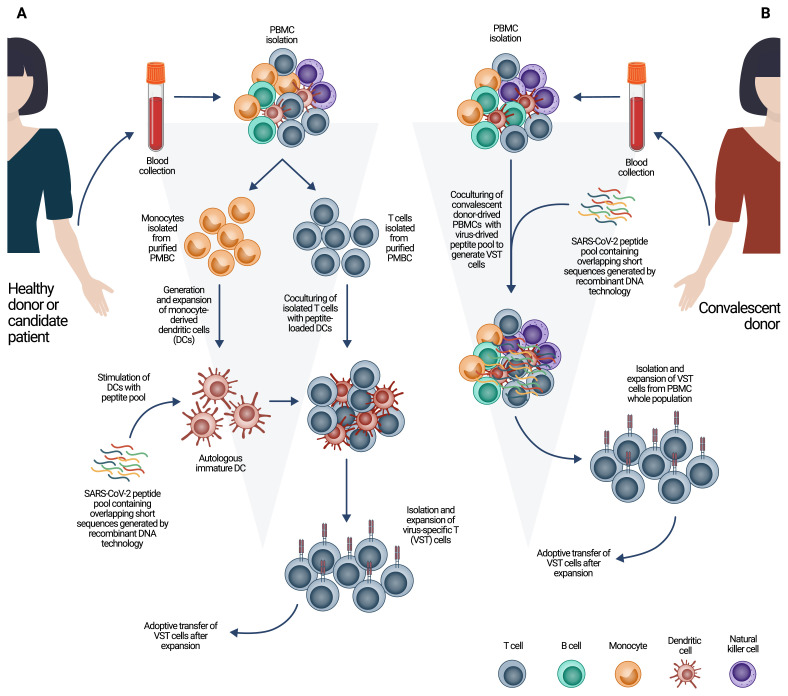
Schematic representation of autologous or allogeneic cell therapy to generate SARS-CoV-2-specific T cells using donor-derived lymphocytes. (**A**) PBMCs from a healthy donor or candidate patient can be isolated for autologous cell therapy followed by monocyte-derived dendritic cells (DC) generation by stimulating of monocytes in an appropriate media to induce DC differentiation. SARS-CoV-2 peptide pool can be established using recombinant DNA technology for stimulation of immature autologous DCs ex vivo. Coculturing of isolated T cells with peptide loaded DCs allows generation of T cells with specific T cell receptors (TCR) that recognize viral antigens. Isolated and expanded virus-specific T (VST) cells could be used for patient with ongoing SARS-CoV-2 infection. (**B**) COVID-19 convalescent donor-derived PBMCs can be cocultured in a feasible environment with SARS-CoV-2 peptide pool to generate T cells specific to SARS-CoV-2 antigens for allogeneic cell therapy. Generation of activated VST cells is followed by isolation and expansion for adoptive transfer of the VST cells.

**Figure 2:**
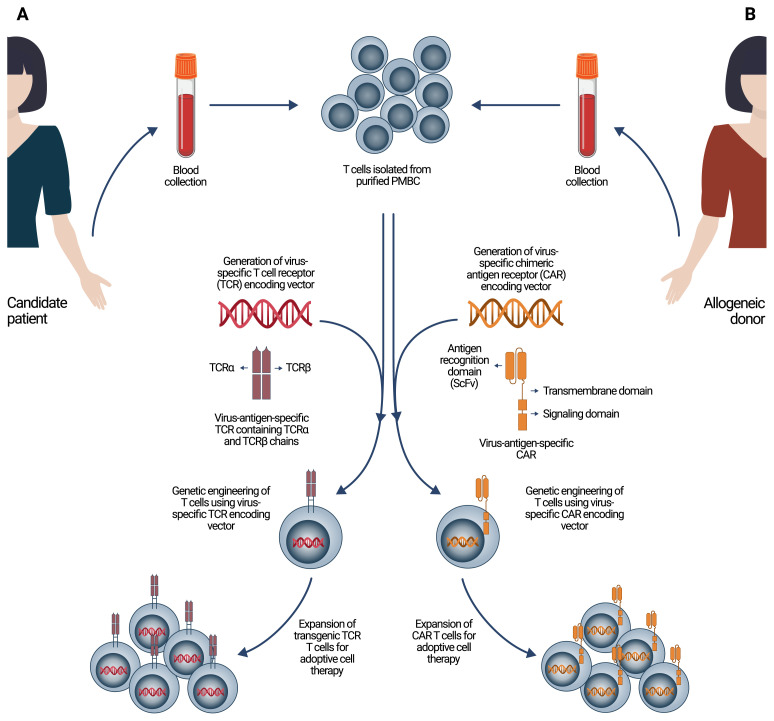
Schematic representation of virus-specific transgenic TCR-T cell and CAR-T cell generation for adoptive cell therapy. (**A**) Isolated T cells from either autologous or allogeneic donors can be genetically engineered using gene vectors encoding virus antigen-specific TCR (**B**) or virus antigen-specific CAR followed by stimulation of the cells for expanding the cell count of transgenic TCR-T cells or CAR-T cells ex vivo to be used for patients.

There have been several approaches to generate virus-specific T cells in general. One strategy allows developing antigen-specific T cells after priming them with APCs that are previously loaded with virus-specific antigens (Figure 1A). Moreover, one clinical study, which is still in Phase I/II clinical trial, has been conducted for COVID-19 in China (NCT04276896) that uses modified DCs carrying COVID-19 targeted genes to generate activated SARS-CoV-2 specific cytotoxic T cells for COVID-19 treatment (Gavriatopoulou et al., 2020; Monzavi et al., 2021). Another approach is to generate virus-specific T cells isolated from peripheral blood mononuclear cells (PBMCs) of convalescent donors activated by using virus-specific peptides followed by enrichment of targeted T cells by immunomagnetic cell sorting mostly based on IFN-γ capturing (Figure 1B). Most of the ACT strategies mainly focused on this type of immunotherapy for COVID-19, since convalescent donors already possess SARS-CoV-2-specific T cells (Keller et al., 2020; Leung et al., 2020; Cooper et al., 2021; Ferreras et al., 2021; Monzavi et al., 2021). Furthermore, genetic engineering methods became popular for ACT to generate virus-specific T cells by sequencing virus antigen-specific TCRs followed by synthesis of the sequence and engineering the TCRs utilizing the vector that contains the sequences (Hayes, 2020; Monzavi et al., 2021). By this method, T cells that have specific TCRs target virus-specific antigens directly are generated and expanded ex vivo to be used for patients (Figure 2A).

Chimeric antigen receptor (CAR) T cells are one of the preferred ones among ACTs in which there are CAR-T cell immunotherapies approved by the Food and Drug Administration (FDA) for different diseases including cancer, autoimmune diseases, and infectious diseases (Seif et al., 2019; Haddadi et al., 2020; Hayes, 2020; Voelker, 2020). CARs are artificial structures containing extracellular regions constituted from a single-chain variable fragment (scFv) of a mAb for target cell detection, together with a part of the intracellular domain for the activation of T cells (Dwivedi et al., 2019; Seif et al., 2019; Monzavi et al., 2021; Zmievskaya et al., 2021). CAR-T cells constitute a major advantage, as it does not require an MHC molecule for antigen recognition. The multistep procedure for developing CAR-T cells for implementation of immunotherapy starts with the isolation of antigen-specific antibodies from the peripheral blood of a convalescent patient, preceded by sequencing of the protein to use it in engineering the sequences and generating CAR vector in an scFv format followed by the viral incorporation or nonviral fusion of CAR genes into the nucleus of the T cells (Dwivedi et al., 2019; Seif et al., 2019; Monzavi et al., 2021; Zmievskaya et al., 2021). Then, the CAR-T cells are multiplied, and the patient is injected with the cell substance (Figure 2B). The major objective of CD8+ T cells is the potential capacity to remove cells and factors that are detected as foreign items and to treat infectious diseases by allowing those cells as favorable CAR-T (Dwivedi et al., 2019; Seif et al., 2019; Chan et al., 2021; Tay et al., 2020). Recently, the method based on the employment of transduced CAR immune cells, as opposed to virus-infected cells, has drawn a scientific interest as an immunotherapeutic approach for COVID-19 (Mathew et al., 2020; Esmaeilzadeh and Elahi, 2021; Monzavi et al., 2021; Zmievskaya et al., 2021).

In addition to the growing variety of therapeutic uses, knowledge acquired from CAR-T cell immunotherapy has enabled scientists to overcome other complications of the disease. The cytokine release syndrome triggered by proinflammatory cytokine over-secretion (e.g., IL-6 or IFNγ) through CAR-T cells stimulated responding infected cell recognition is an important indication (Vardhana and Wolchok, 2020; Zmievskaya et al., 2021). Last but not the least, cytokine release syndrome, characterized by effects such as fever, fatigue, hypoxia, muscle pain, hypotension, and dysfunction in the kidney, is a significant undesirable effect of CAR-T cell therapy (Zmievskaya et al., 2021). Scientific studies are ongoing to overcome those disadvantages, yet enhancement of the method opens the way of treatment options for different diseases and gives rise to developing new strategies for especially emerging infections like COVID-19.

In immunocompromized people, adoptive transferring of virus-specific CD8+ T cells in which cells are derived from donors has proven effective for the management of many different infectious diseases. Yet, some difficulties still exist for adoptive T-cell therapy. Firstly, it is impossible to use allogeneic T cells from unrelated people because of hereditary limitations caused by Human Leukocyte Antigen (HLA) class I, yet the usage is limited to donor-derived T cells and challenges remain for large-scale manufacturing. To this end, graft-versus-host disease (GvHD) might be a significant risk factor for COVID-19 patients after the transfer, thereby suggesting engineered donor-derived cells and banking of HLA- matched lymphocytes may resolve the issue (Cooper et al., 2021; Ferreras et al., 2021; Monzavi et al., 2021). Moreover, by sustained treatment with a particular virus antigen, donor T cells are extended in the culture to obtain an adequate amount of effector T cells to be permeated (Caccamo et al., 2020; Esmaeilzadeh and Elahi, 2021; Monzavi et al., 2021). In cases where CD8+ T cells are depleted and struggling for proliferation and expansion, this is a significant obstacle, as it happens in COVID-19 patients, where the amount of peripheral blood CD8+ T cells is deeply decreased, and CD8+ T cells are functionally depleted. Despite the decreased cell count, aberrant activation of CD8+ T cells is seen and appears to be related to disease severeness. Thus, adoptive transferring of those aberrant cells might raise the aggressiveness and cause damage especially around the lymphocyte-infiltrated lung tissue (Song et al., 2020; Monzavi et al., 2021). Engineering T cells for regulating activation and suppression by creating on-off switches as described in Ellis et al. may increase the efficacy of treatment strategies, but it is not widely applicable for patients with comorbidities such as cancer (Basar et al., 2020; Ellis et al., 2021). Lastly, the so-called “cytokine release syndrome” is a significant side effect of T-cell therapies, a substantial inflammatory reaction that leads to ARDS and multiple organ failure in the patients. A similar condition is also observed in COVID-19 patients, so the toxicity of the therapy would add further complexity such as inflammatory syndromes (Basar et al., 2020, 2021; Caccamo et al., 2020; Esmaeilzadeh and Elahi, 2021).

Besides all possible adverse effects of T-cell therapy that would be seen in COVID-19 cases stated so far, the promising approaches constitute considerable advantages. The cells of human HLA-E-restricted CD8+ T cells have antimicrobial and cytolytic behaviors. These CD8+ T cells, however, also generate IL-4, IL-5, IL-13, and, to a varying degree, IL-10 and TGF-β, in addition to the usual Th1 cytokine IFN-γ (Caccamo et al., 2020; Vardhana and Wolchok, 2020). One study proposes that the use of HLA-E-restricted CD8+ T cells will give many benefits to patients having COVID-19 to improve T-cell immunotherapy, such as the combined ability to destroy infected cells and prevent intracellular infections by minimizing the level of the inflammatory response and reducing adjacent tissue damage, of what is being considered as an essential factor of the pathway (Caccamo et al., 2020; Vardhana and Wolchok, 2020). Another significant feature is the monomorphic antigen recognizing paradigm of HLA-E-restricted CD8+ T cells, which enables the global heterogeneous population to use them (Caccamo et al., 2020). Also, HLA-E-restricted CD8+ T cells are unable to build allogeneic reactions and induce phenomena of graft-versus-host disease. In theory, HLA-E-restricted CD8+ T cells against SARS-CoV-2 can be affordable and easily developed with donations of COVID-19 convalescent allogeneic samples in large quantities, collected and used when needed for serious COVID-19 cases (Caccamo et al., 2020).

There is also a potential to extend the possible adoptive cell therapeutics by addressing the clearance of the infected cells as an alternative strategy to adoptive T-cell therapy. Given the importance of NK cells as effector lymphocytes of innate immunity, the killing tumor and virally infected cells is a potential therapeutic strategy as well. When MHC class I molecules decreased on the surface of the infected cells, NK cells work by either specific identification of virus-related proteins or inhibiting NK receptor signaling. (Golchin, 2020; Zmievskaya et al., 2021).

In brief, using SARS-CoV-2-specific T cells appears to be a reasonable therapeutic approach to treat COVID-19 depending on the adoptive T-cell therapy background in multiple pathological conditions, involving viral infections. Either autologous or allogeneic T cells specific to the virus can be expanded in vitro and administrated to the patient to reconstruct a successful immunity against the virus, and they have provided successful outcomes to treat several viral infections. SARS-CoV-2-specific T cells could be separated and extended using SARS-CoV-2-derived proteins from the bloodstream of convalescent donors and exploited for the treatment of serious COVID-19 cases (Toor et al., 2021). Since using this method for COVID-19 is currently not that common, there is poor knowledge about the effectiveness, toxicities caused by the therapy, and difficulties for using the adoptive T-cell therapy. Significantly, because of genetic limitations (HLA class I), it is not feasible to use incompatible allogeneic T cells, and in vitro expanded T cells could display functional depletion by sustained stimulation to attain necessary cell counts, or transferred T cells could generate cytokine release syndrome, contributing to COVID-19 disease difficulties (Toor et al., 2021). A continuing clinical trial based on preliminary adoptive T-cell therapy for COVID-19 exists, while another prospective clinical trial would follow an innovative solution using SARS-CoV-2-specific T cells from recovered COVID-19 patients for treating patients with elevated respiratory failure (Caccamo et al., 2020; Toor et al., 2021).

## 5. Conclusion

T cell-dependent immune dysregulation has become a major problem in COVID-19 patients leading to an accelerated disease severity depending on the inability of the immune system to defend the body, rapid spread of the virus, and increased anti-inflammatory response. As a matter of fact, elder people or immunocompromized patients who are suffering from different diseases already possess a lower amount of naïve T cells to make an adequate adaptive immune response to a newly occurred infection such as COVID-19. Therefore, adoptive T-cell therapy is a promising treatment option for those patients who struggle to have a T cell response and neutralizing antibody response depending on the inadequate T cell activation or the lack of enough amount of naïve T cells because of an existing medical condition. In conclusion, the creation of emerging adoptive cell therapies, management of critical patients suffering from SARS-CoV-2 infection with maximum efficiency, and, therefore, minimum toxicity would be needed. Thus, the risk of patient outcomes could be minimized, and the benefit of those time-consuming and expensive immunotherapies could be enhanced. This comprehensive point of view would encourage the usage of CAR-T cells in the medical intervention of patients who are infected with SARS-CoV-2, contributing to the improvement of new immunotherapy techniques for patients with extreme COVID-19. In conjunction with other therapeutics to facilitate the advancement of ubiquitous immunotherapy for COVID-19 would be promising.
